# Inhibition of Polo-like kinase 1 reduces beta-amyloid-induced neuronal cell death in Alzheimer's disease

**DOI:** 10.18632/aging.100382

**Published:** 2011-09-13

**Authors:** Bing Song, Korbin Davis, X. Shawn Liu, Hyoung-gon Lee, Mark Smith, Xiaoqi Liu

**Affiliations:** ^1^ Department of Biological Sciences, Purdue University, West Lafayette, IN 47907, USA; ^2^ Department of Biochemistry, Purdue University, West Lafayette, IN 47907, USA; ^3^ Purdue Center for Cancer Research, Purdue University, West Lafayette, IN 47907, USA; ^4^ Department of Pathology, Case Western Reserve University, Cleveland, OH 44106, USA

**Keywords:** Alzheimer's disease, ϐ-amyloid, Cell cycle, Plk1, phosphorylation

## Abstract

Alzheimer's disease (AD) is a progressive and fatal brain disease, but the pathogenesis of AD is still not understood. Aberrant cell-cycle re-entry of neuronal cells is emerging as a potential pathological mechanism in AD. Polo-like kinase 1 (Plk1) is an established regulator of many cell cycle-related events. Interestingly, Plk1 is present in susceptible hippocampal and cortical neurons of AD patients but not age-matched controls. However, whether Plk1 is involved in the pathogenesis of AD remains elusive. In this study, we showed that Plk1 activity is elevated in AD patient brain as indicated by the increased phosphorylation signal of p150^Glued^, a Plk1-specific substrate. Furthermore, we demonstrated that Plk1 is elevated during the cell-cycle re-entry of neuronal cells in an in vitro cell-culture model. Significantly, inhibition of Plk1 kinase activity or depletion of Plk1 by RNAi reduces β-amyloid (Aβ)-induced neuronal cell death. These results validate Plk1 as a possible target for AD therapy.

## INTRODUCTION

Alzheimer's disease (AD) is a fatal brain disease characterized by neuronal inflammation, neuronal cell loss, and decline of memory and recognition [1]. However, as the leading cause for death of dementia, the pathogenesis of AD is still far from being understood. Two hall marks of AD are senile plaques (SPs), which are mainly composed of β-amyloid peptide (Aβ) depositing outside of neuron bodies, and neurofibrillary tangles (NFTs), which are aggregates of hyperphosphorylated tau proteins that bind to microtubules within the neurons [1].

Among all hypotheses for the pathogenesis of AD, cell cycle re-entry has been supported by studies from transgenic mice and patients with AD [2-5]. Increasing evidence points to the cell cycle as a new therapeutic target for AD [6]. Neurons in the adult central neuronal system are terminally differentiated, meaning that they are arrested in the G0 state of the cell cycle. During AD progression, under continued stimuli of proliferation, neurons attempt to exit G0 and re-enter the cell cycle, as indicated by elevation of cell cycle markers and completion of DNA replication [5]. However, terminally differentiated neurons lack the ability to complete the cell cycle and therefore re-activation of the cell cycle machinery will lead to cell death [7]. Thus, one urgent task is to find the stimuli which lead and propel the neuronal cell cycle re-entry and eventually cell death.

Genetic and biochemical experiments have shown that Polo-like kinase 1 (Plk1), the best characterized member of a family of Ser/Thr protein kinases, is a pivotal regulator of the cell cycle [8]. Expression of Plk1 is tightly regulated during the cell cycle; Plk1 is detected at S phase, continues to increase at G2 phase and reaches a peak during mitosis [9]. Consistent with its protein expression level, Plk1 has been shown to be essential for G2/M checkpoint recovery, mitotic entry, centrosome maturation and assembly of bipolar spindles [8]. In addition to multiple mitotic functions, several recent reports have established a link between Plk1 and DNA replication [10-12]. Significantly, Plk1 is highly present in susceptible hippocampal and cortical neurons of AD patients but not age-matched controls [13].

In this study, we show that Plk1 activity is significantly elevated in the hippocampal tissues of AD patients, as indicated by the increased phosphorylation level of p150^Glued^, an established Plk1 substrate [14]. To further dissect the role of Plk1 in AD, we demonstrate that Plk1 protein and its kinase activity are elevated during the neuronal cell-cycle re-entry process induced by Aβ treatment. Furthermore, depletion of Plk1 reduces Aβ-induced neuronal cell death. These results indicate the involvement of Plk1-associated kinase activity in the pathogenesis of AD.

## RESULTS

**Figure 1:**
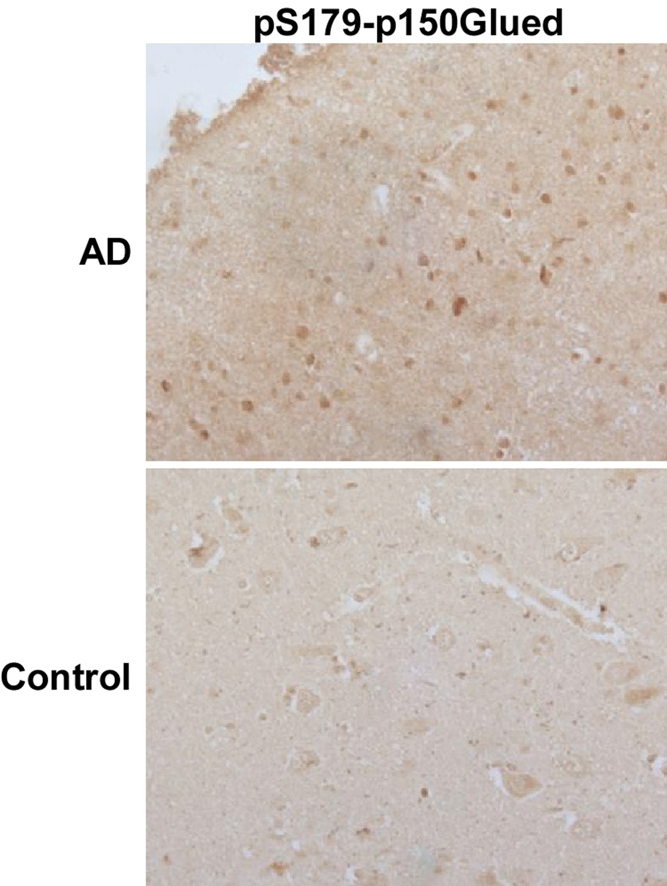
An increased phosphorylation level of p150^Glued^ in AD neurons Hippocampal tissues of AD patients or age-matched controls were subjected to immunohistochemistry staining with phospho-specific antibodies against pS179-p150^Glued^. Plk1 phosphorylates p150^Glued^-S179 specifically [27].

In adult tissues, Plk1 is not present or present at a very low level in non-proliferating tissue cells, such as brain, pancreas and heart [9]. However, Plk1 can be detected in brain tissues of patients with AD [13]. But whether Plk1 is involved in the pathogenesis of AD is still unknown. Considering the fact that Plk1 regulates cell cycle through phosphorylation of its substrates, we examined brain samples of AD patients by immunocytochemistry staining of phospho-Ser179-p150^Glued^. p150^Glued^, the largest subunit of the dynein/dynactin motor complex, plays important roles in many cellular processes, including neurodegeneration [15]. Our published work demonstrated that Plk1-mediated phosphorylation of p150^Glued^ at Ser179 starts at interphase and that the phosphorylation event facilitates nuclear envelope breakdown at prophase of the cell cycle [14]. Phosphorylation of S179-p150^Glued^ was detected in brain tissues of AD cases but not age-matched controls, indicating that not only Plk1 protein is expressed but its kinase activity is also activated in AD patient brains (Figure 1).

**Figure 2:**
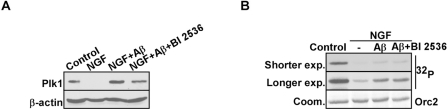
Plk1 expression is elevated in Aβ-treated neuronal PC12 cells (**A**) PC12 cells were differentiated by treatment with NGF for 3d, incubated with Aβ_25-35_ (10 μM) for 24 h in the presence or absence of BI 2536 (10 nM), and harvested for Western blotting with antibodies against Plk1 and β-actin, a loading control. (**B**) Samples prepared in the same way as in (**A**) were subjected to anti-Plk1 IP/kinase assay using GST-Orc2 as a substrate [28], followed by autoradiography. IP: immunoprecipitation.

To further investigate the role of Plk1 in AD formation, we established an *in vitro* cell culture system to mimic aberrant neuronal cell cycle re-entry during the pathogenesis of AD. Rat pheochromocytoma PC12 cells were first fully differentiated to neuronal-like cells by nerve growth factor (NGF) treatment, mimicking the terminally differentiated neurons in adult brains [16]. Then Aβ_25-35_ was then introduced to induce cell cycle re-entry and eventually neuronal cell death [1]. We first monitored Plk1 protein expression level during the process. As expected, Plk1 protein level was abolished after NGF treatment, indicating that PC12 cells were enriched at G0 phase after NGF treatment. Upon Aβ_25-35_ treatment-induced cell cycle re-entry, Plk1 protein level was elevated. When PC12 cells were treated with BI 2536, a Plk1 inhibitor, together with Aβ_25-35_, a slight decrease in Plk1 protein level was observed possibly due to the slowed progression of cell cycle re-entry (Figure 2A). We also performed *in vitro* IP/kinase assays to test Plk1-associated kinase activity in our system. As shown in Figure 2B, Plk1 kinase activity mirrors Plk1 protein level in our system (Figure 2B). These results indicate that Plk1 is expressed and activated during the cell cycle re-entry of neuronal cells.

**Figure 3:**
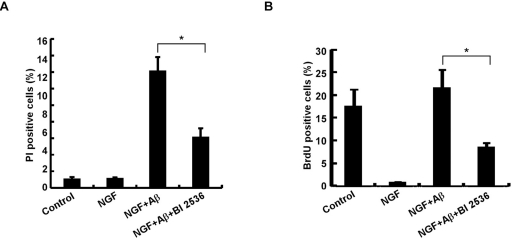
Plk1 is essential for neuronal cell death (**A**) Inhibition of Plk1 reduces Aβ-induced neuronal cell death in PC12 cells. PC12 cells were treated with NGF for 3 d, followed by Aβ_25-35_ or Aβ_25-35_ + BI 2536 treatment for 24h. Cells were then incubated with 10 μg/ml propidium iodide (PI) for 10 min at 37–, washed with PBS, and harvested for immunofluorescence (IF). Cell death was assessed based on the principle that only the nuclei of cells with compromised plasma membranes will be stained with PI. (**B**) Inhibition of Plk1 reduces Aβ-induced DNA replication in PC12 cells. PC12 cells were treated as in (**A**), and subjected to BrdU incorporation assay to monitor DNA synthesis. * P<0.05.

**Figure 4:**
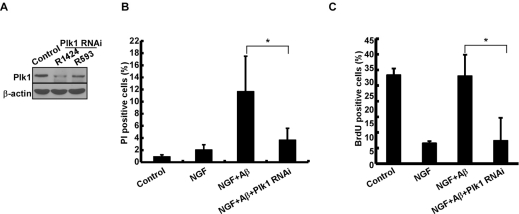
Depletion of Plk1 prevents Aβ-induced cell death and DNA replication in neuronal PC12 cells (**A**) Depletion of Plk1 in PC12 cells. One day after PC12 cells were differentiated with NGF, cells were infected with lentiviruses targeting nt1424 or 593 of Plk1, treated with Aβ_25-35_ on day 4 of post-NGF treatment, and harvested for Western blotting. R1424 and R593 indicate two different targeting sequences on rat Plk1. (**B**) Cells described in (A) were subjected to cell death assay. (**C**) Cells described in (**A**) were subjected to BrdU labeling assay. *P<0.05.

To evaluate the significance of elevated Plk1 level during the cell cycle re-entry process, Plk1 activity was inhibited by BI 2536 treatment. Inhibition of Plk1 significantly decreased Aβ-induced neuronal cell death, indicating that Plk1 promotes Aβ-induced neuronal cell death (Figure 3A). BrdU incorporation assay also showed that DNA synthesis was reduced after BI 2536 treatment, suggesting that Plk1 inhibition prevents Aβ-induced cell cycle re-entry (Figure 3B). Since BI 2536 might also partially inhibit Plk2 and Plk3 activities due to nonspecificity of the drug [17], we performed Plk1 RNAi to test whether Plk1 promotes neuronal cell cycle re-entry and consequent cell death. Knockdown efficiency of Plk1 protein was demonstrated by Western blotting (Figure 4A). Knock-down of Plk1 significantly prevented cell cycle re-entry (Figure 4C) and decreased Aβ-induced neuronal cell death (Figure 4B).

## DISCUSSION

Plk1 is one of the best characterized Ser/Thr protein kinases. Genetic and biochemical studies have shown that Plk1 plays critical roles in many aspects of the cell cycle, such as DNA replication, G2 DNA damage recovery and mitotic entry [8, 10]. Consistent with these functions, the protein expression level of Plk1 starts to increase in S phase and peaks at G2/M [9]. Our published data have shown that Plk1 phosphorylates p150^Glued^ at Ser179 during G2 phase [14]. Significantly, p150^Glued^ is involved in neurodegenerative diseases [15]. In this study, we reported an increased level of phosphorylation of p150^Glued^-S179 in AD brains compared to age-matched controls, indicating that the elevated Plk1 protein in the neurons of AD patients is activated and able to phosphorylate its known substrates. Furthermore, our finding suggests that Plk1 might be a feasible therapeutic target for AD, as several small-molecule inhibitors of Plk1 are under clinical trials [8].

To investigate the role of Plk1 in depth, we established an *in vitro* cell culture model to mimic the neuronal cell cycle re-entry during the pathogenesis of AD. We found that Plk1 is expressed and activated during the neuronal cell-cycle re-entry process induced by Aβ-treatment. Aberrant cell cycle re-entry has been characterized as an early event of AD progression [1], and several cell cycle markers, such as cyclin D, cdk4, cyclin B, phospho-H3 and p27, are present in terminally differentiated AD neurons [18]. However, these proteins are detected in different spatial patterns as they normally do [19-21], supporting the notion that these cell cycle regulators might have novel functions during the pathogenesis of AD. For that reason, we acknowledge that we cannot exclude the possibility that Plk1 might also have a novel function in AD. However, the IHC staining data of phospho-p150^Glued^-S179 suggest that Plk1 kinase activity is activated in AD and that Plk1 phosphorylates its documented substrates in normal cell cycle. Significantly, our data demonstrate that Plk1 inhibition or depletion slowed cell cycle progression (Figure 3B, 4B) and reduced Aβ-induced neuronal cell death (Figure 3A, 4A).

Furthermore, activation of the mTOR pathway has shown to be involved in age-related diseases, such as Alzheimer's disease. Two of the downstream targets of mTOR are the ribosomal p70S6 kinase (p70S6K) and the 4E-binding protein 1 (4E-BP1) [22]. Recent study shows that Plk1 inhibition decreases phosphorylation of these two mTOR downstream targets, thus inhibits mTOR pathway [23]. And inhibition of mTOR pathway slows down aging thus age-related diseases in varies species [22]. Plk2, which is another polo-like kinase member, has also been linked to mTOR pathway regulation [24]. Our findings support and further suggest that prevention of cell cycle re-entry by inhibition of Plk1 might be a promising strategy for AD therapeutics. Understanding the initiation factor of neuronal cell cycle re-entry will help open a path to pursue the prevention or cure of AD.

## MATERIAL AND METHODS

### Cell culture

Rat pheochromocytoma PC 12 cells were cultured in RPMI-1640 medium (ATCC, 30-2001) with 10% of heat-inactivated horse serum (Sigma, H1138) and 5% of fetal bovine serum (Atlanta, S11550). To induce differentiation of PC12 cells, we seeded the cells on the plates coated with Poly-L-lysine (Sigma, P4707) for 24h, then replaced the complete culture medium with differentiating medium (RPMI-1640 supplemented with 1% heat-inactivated horse serum and 100ng/mL Nerve Growth Factor-7S) with medium change every other day. Cells were treated for 3 days to be differentiated into neuronal-like cells [16].

### Aβ

Previous reports have shown that Aβ_25-35_ and Aβ_1-40_ have a comparable biological effect on inducing cell death [25], thus Aβ_25-35_ was used in this study. Aβ_25-35_ (Sigma, A4559) was dissolved in sterile water to a stock concentration of 1 mM. To prepare aggregated Aβpeptides, stock Aβwas mixed with same amount of PBS, and incubated for 3 days at 37°C. Cells were treated at 25 μM for 24 h for various assays.

### Kinase assay

For *In vitro* IP/kinase assays, total cell extracts were immunoprecipitated (IPed) with Plk1 antibodies (Santa Cruz, sc-17783), and the IPed proteins were subjected to kinase assays with GST-Orc2 as substrate. Kinase assays were performed in TBMD buffer (50 mM Tris, pH 7.5, 10 mM MgCl_2_, 5 mM dithiothreitol, 2 mM EGTA, 0.5 mM sodium vanadate, 20 mM *p*-nitrophenyl phosphate) supplemented with 125 μM ATP and 10 μCi of [γ-^32^P]ATP at 30°C for 30min. After the reaction mixtures were resolved by SDS-PAGE, the gels were stained with Coomassie Brilliant blue, dried, and subjected to autoradiography.

### RNA interference

To specifically deplete Plk1, lentiviruses were generated with the targeting rat sequences AGATCACTCTCCTCAACTATT and CAACAAAAGTGGAATATGAAG [26]. Lentivirus infection was performed in the presence of Polybrene (10 μg/ml) and HEPES (10 mM).

### BrdU labeling assay

BrdU-labeling assays were performed with a kit from Roche according to the manufacturer's instructions.

### Cell death assay

Cells were incubated with 10 μg/ml propidium iodide (PI) for 15 min at 37°C and washed with PBS, followed by DAPI staining.

### Western blotting

Cell lysates were resolved by SDS-PAGE and detected by Western blotting with antibodies against β-actin (Sigma, A5441) and Plk1 (Santa Cruz, sc-17783).

### Tissue

Tissues were prepared and stained as described previously [5].

### Statistics

Data were analyzed by one tailed unpaired student's t-test.

## Abbreviations

AD: Alzheimer's disease
BrdU: 5-bromo-2′-deoxy-uridine
DAPI: 4′6′-diamidino-2-phenylindole
IF: immunofluorescence
IHC: immunohistochemistry
IP: immunoprecipitation
NGF: Nerve growth factor
Plk1: polo-like kinase 1
RNAi: RNA interference
Aϐ: beta-amyloid
